# Mobilizing morality: how caregivers in Vietnam handle the challenges of daily diabetes care

**DOI:** 10.1186/s12889-023-16691-8

**Published:** 2023-09-07

**Authors:** Dieu Bui Thi Huyen, Ai Nguyen Thi, Dung Vu Thi Kim, Hieu Le Minh, Tine Gammeltoft, Amalie Rørholm Vestergaard

**Affiliations:** 1 Thai Binh University of Medicine and Pharmacy, Thai Binh, Vietnam; 2 Department of Anthropology, University of Copenhagen, Copenhagen, Denmark

**Keywords:** Type 2 diabetes, Caregivers, Informal care, Vietnam

## Abstract

**Background:**

As a chronic disease, type 2 diabetes (T2D) often involves long-term care obligations for patients’ family members. Understanding the socially and culturally specific challenges that family caregivers face and how they cope with them is crucial in developing targeted and effective interventions to support both caregivers and patients with T2D. This research examined family caregiving for people with T2D living in rural northern Vietnam. Although there is a growing literature on family support in Vietnam, little is known about the personal experiences of family caregivers for people with T2D. This paper seeks to fill this gap revealing some of the challenges and coping strategies of family caregivers to people with T2D.

**Methods:**

This qualitative study is based on ethnographic research using primarily semi-structured interviews with 21 caregivers to a person with T2D in Vietnam. The research was conducted in 2022 by a Vietnamese-Danish research team. Each interview was voice-recorded, transcribed verbatim and thematically coded.

**Results:**

Four major challenges emerged from the analysis: physical health concerns, psychological exhaustion, economic burdens, and lack of support. Caregivers expressed motivation to overcome these challenges as they felt a deep sense of responsibility towards their family member with diabetes. The primary caregiver’s sense of responsibility toward their family would often cause them not to share the burdens from caregiving with other family members to avoid burdening them as well. However, negative experiences from caregiving were decreased and positive feelings increased in the instances where caregiving was shared between multiple family members.

**Conclusion:**

While family members expressed motivation to take care of the patient because of moral obligations, some caregivers, specifically primary caregivers, did not want to burden other family members with care tasks and were reluctant to ask for assistance. For families who did share the caregiving tasks among several family members, some of the negative sentiments associated with caregiving were diminished. Having multiple members of a family forming a caregiving community thus motivated people in handling care challenges.

## Introduction

Across the world, the prevalence of diabetes mellitus is increasing as the number of people diagnosed is expected to rise from 537 million in 2021 to 783 million in 2045 [1]. Like many chronic conditions, diabetes requires complex medical management and challenging daily care, which requires following regimented eating plans, monitoring blood sugar levels, organizing daily medications, and coordinating medical care [2]. Along with the rise in people diagnosed with diabetes, and the fact that self-care is currently promoted as a solution to burdens on health care systems [3], the responsibility for day-to-day disease management increasingly moves from health-care professionals to individuals and families. American and European guidelines for diabetes management are often not followed in low and middle-income countries, due to resource constraints [4]. The management of T2D is thus a challenge in low and middle-income countries because of the difficulty of access and affordability in health care systems, and because of the lack of health care workers and clinical information [5].

In this article, the term family caregiver refers to individuals who provide primary care to a family member with diabetes. It could be a spouse, adult child, or any other relative responsible for assisting in personal care and mobility, performing household duties, solving financial problems, or help inject insulin.

In recent years, studies have shown that caregiver involvement in patients’ diabetes care is associated with more successful self-management and better health outcomes [6–9]. Though family caregivers play a pivotal role in the well-being of the T2D patient, research shows that caregivers in Thai Binh lack knowledge on how to help and support care recipients in managing their diabetes [10].

From 2011 to 2021, the prevalence of T2D among adults in Vietnam increased from 3.2% to 6.1% [11]. However, the Vietnamese health care system is not fully equipped to provide care for people with non-communicable diseases, as primary health care institutions often do not have the necessary capacity and technology to support patients with T2D and other NCDs in managing their disease [12]. In other low- and middle-income countries, studies have similarly shown large unmet needs for diabetes care in general [13, 14].

In Vietnam, the health care system consists of four levels: national, provincial, district and commune. Services for prevention, diagnosis and treatment of diabetes are difficult to access, especially at the commune level [15]. Although social health insurance in Vietnam covers direct medical costs, other costs such as transportation, food, over-the-counter medication, and traditional medicine are covered by the patient and/or their caregiver(s) [16]. A report from the Vietnamese Ministry of Health showed that the rate of patients treated by self-medication account for 73% [17]. A poor household spends 47.12% on average of their total income on diabetes, with the highest spending being related to medications [18]. Although caring for sick loved ones can lead to negative impact on caregivers, research has shown that Vietnamese caregivers accept their role because of moral and social obligations [19].

While the literature on people with T2D has expanded, studies on the experiences of family caregivers are highly limited. To our knowledge, no qualitative study has yet investigated the challenges associated with caring for people with T2D in Vietnam. To improve quality of care, it is essential to understand the specific challenges that caregivers are facing.

To address these issues, this qualitative study focuses on the caregiver’s point of view. Through analyzing the caregivers’ experiences in caring for their family member with diabetes, this study aims to explore what challenges caregivers face and how they deal with them. A comprehensive and in-depth understanding of the family caregiver’s struggles can help health care providers in planning programs aiming to support caregivers.

## Methods

### Research setting and sample

The study was conducted in a rural area of Vietnam’s Thai Binh province. Thai Binh is located in the northern part of Vietnam with approximately 2 million residents of whom the majority live from wet-rice cultivation. The research was conducted under the auspices of a larger interdisciplinary and capacity-building research project investigating informal care for people with T2D. The overall project combines epidemiological and ethnographic methods, and the present study was conducted as a part of the project’s ethnographic component [20]. The ethnographic research aimed to understand how people with diabetes and their informal caregivers experience and respond to the disease, with a special focus on the challenges they face. As always in ethnographic research, emphasis was placed on understanding T2D as the affected individuals themselves understand it, giving careful attention to the social conditions and cultural meanings through which the disease emerges as a local problem. The ethnographic research was conducted as an extended case study [21] involving 27 people with diabetes and their informal caregivers. In the first stage of the ethnographic study we worked mainly with people with diabetes themselves (for details on sampling for the first stage of the ethnographic research, see article [21]), whereas the second stage of the data collection, carried out from March to June, 2022, included semi-structured interviews with family caregivers. We conducted interviews with 21 caregivers, meeting with the people that patients with T2D themselves defined as their most important caregivers. For socio-demographic characteristics of the caregivers, see Table 1.

**Table 1:** Characteristic of caregivers (*n* = 21)

Varriable		Number	Percentage
Gender	Male	10	47.6
	Female	11	52.4
Age in years	< 60	7	33.3
	60–79	12	57.2
	≥ 80	2	9.5
Relationship with the patient	Spouse	15	71.4
	Daughter/son/daughter-in- law	4	19.1
	Others	2	9.5
The forms of support	Nutrition	7	33.3
	Treatment	4	19.1
	Finance	6	28.6
	Emotion	7	33.3
	All	5	23.8
Duration of caring	1–5 years	0	0
	6–10 years	12	57,1
	> 10 years	9	42,9

### Data collection

To guide the interviews, a semi-structured interview guideline was developed. The guideline included a set of key issues to be investigated in the interview, with the order of the questions varying depending on the context of each interview. The main topics were: caregivers’ life situations, the nature of the care provided, their own perceptions of their roles as caregivers, the challenges they faced, and their coping strategies, motivations, and social and familial support.

The interviews were performed by five Vietnamese researchers and one Danish researcher and were conducted face-to-face in the home of the participants. Most interview sessions had a duration of approximately two hours. The interviews were recorded by an mp3 recorder and later transcribed. All interviews were conducted in Vietnamese. If the family member with diabetes was present in the house at the time of the interview, the researcher also asked to meet and talk to them. After each interview, the researchers wrote up detailed ethnographic fieldnotes, including observations, conversations, and key events. The fieldnotes were shared with other research team members and used as the basis for on-going development and refinement of the research questions, thereby enhancing the rigor of the research. In this research, ethics was not merely a matter of formal approval, but also a constant concern during fieldwork where the researchers did their utmost to engage with research participants in careful and considerate ways, so as to not add further worries to already overburdened households. All ethnographic data was stored on an online platform and accessible to all team members.

### Data analysis

The interview transcriptions were systematically coded based on a coding set developed from the original research questions and the researchers’ field notes. The main coding categories were: caregiver’s life; work; the diabetes situation of patients; the main care activities; challenges in care; feelings about care; support from others. Each researcher coded the transcriptions and fieldnotes from the cases for which he/she held responsibility. When coding was completed, all researchers read through the codings, using the coded transcriptions as the basis for write-up of articles. The research participants did not read the transcriptions or the coded data.

## Results

The sample included 11 men and 10 women. The mean age of the participants was 63.9 ± 15.4 years (ranging from 29 years old to 82 years old). The majority of the T2D patients were cared for by their spouse (71.4%).

In the following, we will first present the challenges that caregivers reported. Next, we move on to examine how they would overcome them. Four main challenges for caregivers were identified from the ethnographic material: physical health concerns, psychological exhaustion, financial burden, and lack of support.

### Caregiving challenges

#### Physical health concerns

Caregivers reported several challenges in relation to physical health. Firstly, caregivers worried about the physical health of the diabetes patient. One caregiver, a wife to the patient, articulated her worry about severe hypoglycemia which can lead to death if not detected in time: *“I am so worried. Hypoglycemia mainly happens at night, so I have to pay close attention. If he is in a lethargic state, and there is no response, I must immediately call my child to take him to the hospital, many times already. Several times, if I did not detect this, he would have died”*.

Watching their family members’ health deteriorate is cause for concern and fear among the caregivers. Another wife to a patient said: “*My biggest fear is that he has to go to the hospital. Every time I see the emergency room, I am very scared because he has been in the hospital for the whole year; I have to stay up all night for a long time. Now he gets tired even after only sitting for a while, so he can't do any heavy work”.*

Most of the patients in the study struggled with other chronic diseases such as hypertension and cardiovascular disease, and some had severe complications from diabetes such as amputation or retinal complications. Therefore, supporting the patients required that the caregivers themselves were in good health. However, 15 out of 22 caregivers were 60 years old or older, including three caregivers who were past 80 years old. Additionally, most of them had one or more chronic diseases, and they often needed care from others themselves.

Many of the caregivers in this study said that they felt run down and that their health status was not adequate for supporting their family member with diabetes. Findings of the study therefore indicate that caregivers are challenged by coping with their own health problems as well as the patient’s.

Putting these challenges into words, a 73-year-old wife said: “*I often have headaches, dizziness when walking, and vestibular disorders. Additionally, my knee hurts and walking is very painful. He (patient) is tall and big, I have to brush his teeth, wash his face and give him a bath every day. Every day, I'm the only one who takes care of him. I feel very tired”.* The family caregivers thus feel responsible for and worry about both the family member’s physical health as well as their own, as their ability to care for the person with diabetes depends on their own physical abilities.

#### Psychological exhaustion

Another challenge that became apparent when talking to the caregivers were their experiences of psychological exhaustion. Many reported that the development of complications from diabetes, changes in physical abilities and the emotional state of the family member with diabetes had negative effects on them.

Most of the caregivers expressed high levels of anxiety and fear, especially when their loved one suffered from severe complications from diabetes. A husband shared his worries: *“When she couldn't eat or fainted, I was worried, I was sad. Due to necrosis, now both of her legs have been amputated. I feel very worried. She has gone to the hospital several times a year. The hospital makes me feel scared. Caring for her there is extremely hard work.”* A wife said: *“Before he was paralyzed, he also supported me with many things, now he lies like that and can't walk. I'm so sad and worried. I just hope he will be able to walk again so I will not have such a hard time”.*

Stress and fatigue in caring for the patient can lead to negative emotional experiences in both the patient and their caregivers. A husband of a patient responded: *“I have many illnesses and still have to support her. It's so hard for me, sometimes I'm so tired that I get angry, I curse her, it's very stressful”.* Another wife-caregiver said: *“I'm tired after working hours when I come home. I also do all things at home, he doesn't understand, he always complains and nags, so sometimes I feel extremely exhausted.”* This makes it apparent that the psychological exhaustion that caregivers experience sometimes strain their relationship with their family member and can push them into behaving in a way towards them that they otherwise would not.

#### Economic burdens

Some caregivers reported that they had to give up work opportunities because they were taking care of their family member with diabetes. Moreover, caregivers reported difficulty in maintaining a full-time job and described having to give up work activities when these did not fit with their caregiving schedule. Caregiving could therefore have a direct negative impact on a family’s finances. One wife described her work situation in these words: “*I would prefer to work in a company or another job. The job I have now is manual labor. It is very hard but the time is flexible. I have to take care of him so I cannot take another job.”* Another caregiver explained that she could not balance caregiving with full-time employment, and as a result, she had to leave her job: *“Years ago, I went to work abroad, but my father was sick, so I had to return home. I can't work because I have to take care of my father and my two young children.”*

At the same time, the study showed that in rural areas in a middle-income country like Vietnam, the medical expenses for people with diabetes in general, and people experiencing complications from diabetes in particular, are a significant burden for them and their family. One husband said: “*She has a health insurance, but the medicines for diabetes covered by the health insurance are not good enough. So we must buy extra medicines ourselves. Also, besides the diabetes she has many other diseases. So the total cost of her treatment in recent years has been about 500 million* (about USD 20,950) *while she and I have only 3 million* (about USD 126) *in pension per month. I used to work in pig farming, but she always had to go to the hospital and I have to take care of her, so I cannot work like I used to.”*

Another husband said*: “Money is very important, because I don't have a lot of money, so I can't buy good medicine for treatment and prevention of complications for hee. My economy can only meet about 60% of the treatment needs of the disease.”* Economic burdens thus contribute to further psychological stress, as caregivers feel they must earn the money they need to pay for the medical expenses. However, this often proves difficult, as tending to a steady job and caring for their family member are difficult to combine.

#### Lack of support

The last main challenge identified was a lack of support from other family members or social networks. The research revealed that people who are solely responsible for the care of a family member with diabetes are prone to experience loneliness in their caregiving.

Participants gave numerous explanations as to why other family members were unable to contribute to the caregiving. Spousal caregivers most often explained the lack of support with the work demands and family obligations that their children or grandchildren might have. This would cause elderly couples to resist dependency on their children and to not want to ask for their assistance. One wife said: *“Normally it's only me taking care of him, when he's in the hospital as well as at home, from eating, and bathing to learning to walk. The children have only had to take care of him when I was isolated due to Covid. When I ended the isolation, I took care of him again. The children have to go to work and take care of their families, and they also don't have the money to support us. The costs for treatment in hospital and medicine are our own expense.”*

Adult children, who were also a major source of care, were often the primary or sole caregivers when the other parent had passed away or their physical health was not adequate for caregiving. Adult children would also emphasize the conflicting obligations that their other family members, particularly siblings, had to fulfill as a reason for the lack of assistance in caregiving. One son said: *“There is only me and my wife. I have a brother and a sister but mostly I do everything for him* [the father]. *I even had to quit my job to stay at home to take care of him. My siblings also have children and go to work, so they can't take care of him often.”*

Being solely responsible for a family member's health places all of the above-mentioned challenges on one family caregiver, causing feelings of loneliness and isolation. Yet the caregivers resisted burdening other family members.

### Caregiver responses to challenges

#### “It is my responsibility”

Caring responsibilities can be overwhelming and lead to stress, worry, financial devastation, and feelings of loneliness, but caregiving responsibilities can also lead to feelings of love, generosity, and can strengthen family ties. Many caregivers expressed gratitude for the opportunity to provide care and to be a part of this journey in their family member’s life. Notably, they saw it as an important part of their familial role towards each other.

Caregivers, especially spouses, usually perceived the role of taking care of their family member as part of their destiny as a spouse and as a part of life. In Vietnam, spouses accompany each other through old age and express a sense of responsibility to take care of each other throughout life. Many participants in the study described the mutual care between spouses as a better approach to receiving care than receiving it from one’s child. A husband explained: *“As husband and wife, we must rely on each other. That is the obligation. If she's sick, I have to take care of her. When I feel tired, I'll overcome it myself. I try to encourage her.”* Another husband described his sense of responsibility in these words: *“I have had cardiovascular disease for many years, and I was bedridden a few years ago, only she took care of me. Now that I'm better I take care of her again, that's my responsibility. Even if it's hard, you have to try.”*

A wife also described feeling pressures from caregiving, but said that she would never complain about it, as caring for each other as spouses is essential and is also a source of positive feelings: *“Sometimes there is a lot of pressure in caring for him, I do not dare to complain to anyone. When I see he is in better health, I also feel happy. The caring between husband and wife is best. Caring for each other helps us to always have comfortable thoughts, not thinking anything negative. If you feel happy, anything can be overcome.”*

Adult children often described the responsibility to care for their parents in similar terms. One son said: *“My family lives with him so I have responsibility to take care of him. In addition to taking care of my father, I also have to take care of my blind mother-in-law because there is no one else to take care of her. This is the responsibility of children to their parents.”* This participant thus emphasized an inherent responsibility that is part of adult children’s familial role to which they must live up. Caregiving then is considered a part of family life and seen as obligations that strengthen family ties: familial roles are cemented through caregiving. Mobilizing the feeling that “it is my responsibility” seemed to help caregivers to keep up spirits.

### Seeking assistance from others

While several caregivers described being solely responsible for the care of their family member with diabetes, some would share this responsibility with other family members and people in their social network. One caregiver, whose husband had diabetes, described how all her children and grandchildren helped with the care: “*I am only strong enough to cook, feed, wash his clothes every day, but to change diapers and give a bath or inject medicine, our son must do it. I am not strong enough. Our son, daughter, and grandchildren, they all support by giving us money and rice. They take good care of their father.* Another wife of a patient described receiving economic support from their children: *“Economic support for us is provided mainly by our eldest daughter, she pays our hospital fees and medicine. Our two sons-in-law also support us in many ways. They all take really good care of their dad.”*

Both wives emphasized how their children would take good care of their father, stressing that this was a source of happiness to them. This also shows how caregiving strengthens family ties for all family members when everyone participates and creates a sense of security in each other as they come together to support a family member. The results showed that when caregiving tasks were shared, feelings of loneliness and other negative emotions were less pressing for the caregivers while the positive feelings associated with taking care of a family member were enhanced.

## Discussion

This study fills an important gap in the qualitative literature on the experiences of family caregiving towards people with diabetes in Vietnam, and the findings have important implications for future designs and contents of intervention programs. This study contributes to the literature on the challenges of chronic disease caregivers, not only in the Vietnamese context, but also in other similar contexts.

A previous article from this project showed that diabetes patients experience existential vulnerability as the disease threatened their health, caused them feeling anxious to burden their loved ones with their disease, and threatened their ability to belong in the larger community [22]. As this indicates, diabetes affects not only the lives of those suffering from the disease but also the lives of the family members who care for them. In many ways caregivers also experience this existential vulnerability as their lives become entangled with their family member’s disease.

Other studies document how caring for chronically ill family members or significant others at home influences multiple aspects of caregivers’ lives [23, 24]. Families are often the primary source of care and support for older relatives and patients with chronic diseases, contributing services that would cost hundreds of billions of dollars annually if they had to be bought [25]. Family caregivers in Vietnam fulfill multiple important roles in the care of patients with chronic diseases—roles that are often considered an integral part of family life and its responsibilities [19, 26].

As presented in the results, the study revealed four major challenges for family caregivers: physical health problems, psychological exhaustion, economic burdens, and lack of support. The health status of their family member with diabetes stood out as a major concern to family caregivers, especially in case of diabetes complications such as amputation or vision loss. Severe comorbidities and complications have previously been proven to contribute to social, psychological, physical, emotional, and practical challenges for caregivers [27]. Previous studies also show that the health status of people with chronic disease has direct impact on their caregivers’ own physical health [28, 29]. This aligns with the findings in this study, where family members reported worrying about the patient’s health and experiencing challenges with their own health when caring for their loved one.

The second challenge of family caregivers in this study was psychological exhaustion. Studies have shown that caring for people with diabetes long-term makes caregivers susceptible to health problems such as sleep difficulties, pain, headaches, and chest pain [30]. Some studies indicated that caregiving burdens heighten risks of depression, anxiety, and poorer quality of life [31]. Our findings thus resonate with previous studies, as the participants described experiencing a heavy burden on their mental health and their everyday emotional state.

The struggles that participants experienced with maintaining their household economy are also in line with previous studies. Struggling with the costs of treatment, caregivers in our study reported that the caregiving tasks hampered their ability to maintain a steady job and made earning a living difficult. Similarly, previous studies have shown that caregiving might interfere with work, leading to lower performance and fewer promotions and thus leading to caregivers earning a lower wage [32, 33]. Financial issues are serious concerns for caregivers both in developing countries and among vulnerable populations in developed countries [34–37].

Finally, lack of support from other family members sometimes placed all the burdens from caregiving on one person. This caused considerable distress and negative feelings. This is consistent with previous studies as well [38–40]. As caregivers did not want to burden and rely on other family members, many caregivers were shouldering the care tasks themselves. For female caregivers, this often meant adding to an already existing care burden. Because of gendered social norms the general caregiving roles in the household are ascribed to female family members[41]. This also meant that the female caregivers in this study often took care of several people in the family. Besides taking care of people with diabetes, they may also have had to take care of children, grandchildren, and even other elderly people in the family, as well as undertaking general household chores. Gender inequality in caregiving has been described in previous studies on family caregiving[42].

Despite facing many challenges and experiencing a heavy burden with the care tasks, caregivers in our study also reported positive sentiments about their caregiving role. Most caregivers regarded caring for their loved one as an important responsibility. In northern Vietnam, it is the norm to live with one’s family and be taken care of by family, when growing old and/or becoming ill. Spouses and children often provide primary care because of their immediate access to the patient either by living with them or living close to them [43, 44]. In this study, caregivers described the diabetes caregiving role as a natural extension of roles taken on as part of living in a household as a family together. This resonates with long-standing Vietnamese family values which are rooted in the triple teachings of Buddhism, Confucianism and Taoism. These values define the needs of the family as a whole as more important than the needs of any individual family member. Family obligation is a critical component of Vietnamese culture, which refers to an implicit societal expectation of mutual support among family members [45, 46]. Caregivers in this study highly emphasized the traditional values of “filial piety” (*lòng hiếu thảo*), as they described their responsibility towards their parents. Filial piety indexes the values of love, compassion, and sacrifice between children and parents, where a child is educated from a young age about their “responsibility/obligation” (*trách nhiệm/nghĩa vụ*) for respecting and taking care of their parents, particularly when they become older and sick [47]. Taking care of their parents means to “return” the love and sacrifices they received from their parents when they were younger [48, 49]. Previous studies conducted among family caregivers for people with dementia in Vietnam have come to similar results, emphasizing the motivational aspects of familial obligations [19, 50].

To face caregiving challenges, caregivers in our study thus mobilized these moral feelings within themselves when describing their inherent familial responsibility towards a family member. Caring for their family member strengthened these family ties and caused positive feelings as a spouse or a child felt a sense of fulfillment of their familial role. Another source of motivation for caregiving, which proved more difficult for some to utilize, was to ask for support from other family members. Previous research on family caregiving for patients with chronic diseases shows that when families include more than one family member in caregiving tasks it has a variety of positive effects for the primary caregiver, e.g., decreasing the overall burden and feelings of loneliness, and maintaining the caregiver’s resilience [51–54].

Yet, the results of this study showed that many primary caregivers, mostly spouses, tried to avoid burdening other family members with caregiving tasks. A previous article from this project used the concept *disease diplomacy* to describe how diabetes patients in Thai Binh tend to their disease on an everyday basis [21]. Disease diplomacy refers to the ways in which diabetes patients attempted to balance attentiveness to and discretion about their disease. This meant managing it as best they could with the limited information and resources they had, while not attending too much to the disease to avoid negative emotions and choosing not to talk with others about the disease to avoid burdening them. Similarly, caregivers in our study took their responsibilities towards their sick family member very seriously, while being reluctant in burdening others with the negative emotions from the disease. Ironically, it is the same familial moral values that motivate caregivers when they face challenges in caregiving tasks that also prevent them from seeking assistance from other family members. As seen from the results, caregivers do not want to burden other family members, whom they assume have other important obligations that they should focus on. As no individual family member is considered more important than the whole, much needed assistance is rarely asked for.

## Conclusions and recommendations

This study has shown that caregivers for people with T2D face many challenges among which we highlighted four in particular; physical health concerns, psychological exhaustion, economic burdens, and lack of support. As family members defined caregiving as a moral obligation, caregivers were positively motivated in their care tasks as caregiving made them feel fulfilled in their family roles and strengthened family ties.

At the same time, many family caregivers experienced loneliness if they did not share the caregiving with other family members. As caregivers did not want to burden other family members, they were often reluctant to ask for assistance. For families who did share the care tasks among several family members, some of the negative sentiments associated with caregiving diminished and positive feelings were enhanced as familial feelings were further consolidated. Having multiple members of a family being part of caregiving thus motivates people in handling care challenges.

These findings point to the importance of informing family members about the complexities of care associated with T2D. It is of utmost importance that family caregivers understand and are well-informed about the complexities of caring and what the caregiver burden can accumulate to. If people become better informed on the complex caregiving that other family members undertake, they might be motivated to offer more support. Thus, information about the disease targeted towards the whole family is crucial to maintain a sense of moral obligation to care for both the family member with diabetes and their primary care provider.

## Data Availability

The datasets generated and/or analysed during the current study are not publicly available but are available from the corresponding author on reasonable request.
